# A synthetic system for expression of components of a bacterial microcompartment

**DOI:** 10.1099/mic.0.069922-0

**Published:** 2013-11

**Authors:** Frank Sargent, Fordyce A. Davidson, Ciarán L. Kelly, Rachelle Binny, Natasha Christodoulides, David Gibson, Emelie Johansson, Katarzyna Kozyrska, Lucia Licandro Lado, Jane MacCallum, Rachel Montague, Brian Ortmann, Richard Owen, Sarah J. Coulthurst, Lionel Dupuy, Alan R. Prescott, Tracy Palmer

**Affiliations:** 1 College of Life Sciences, University of Dundee, Dundee DD1 5EH, Scotland, UK; 2 Division of Mathematics, College of Art, Science & Engineering, University of Dundee, Dundee DD1 4HN, Scotland, UK; 3 School of Computing, College of Art, Science & Engineering, University of Dundee, Dundee DD1 4HN, Scotland, UK; 4 Ecological Sciences, The James Hutton Institute, Invergowrie, Dundee DD2 5DA, Scotland, UK

## Abstract

In general, prokaryotes are considered to be single-celled organisms that lack internal membrane-bound organelles. However, many bacteria produce proteinaceous microcompartments that serve a similar purpose, i.e. to concentrate specific enzymic reactions together or to shield the wider cytoplasm from toxic metabolic intermediates. In this paper, a synthetic operon encoding the key structural components of a microcompartment was designed based on the genes for the *Salmonella* propanediol utilization (Pdu) microcompartment. The genes chosen included *pduA*, -*B*, -*J*, -*K*, -*N*, -*T* and -*U*, and each was shown to produce protein in an *Escherichia coli* chassis. In parallel, a set of compatible vectors designed to express non-native cargo proteins was also designed and tested. Engineered hexa-His tags allowed isolation of the components of the microcompartments together with co-expressed, untagged, cargo proteins. Finally, an *in vivo* protease accessibility assay suggested that a PduD–GFP fusion could be protected from proteolysis when co-expressed with the synthetic microcompartment operon. This work gives encouragement that it may be possible to harness the genes encoding a non-native microcompartment for future biotechnological applications.

## Introduction

Compartmentalization of biochemical processes is an essential feature of all cellular systems. The occurrence of membrane-bound organelles is well documented for eukaryotic systems and it is well established that Gram-negative bacteria house specialized biochemical processes in their periplasms. In recent years, however, it has become increasingly clear that proteinaceous subcellular compartments akin to organelles are utilized by some prokaryotes and that they serve to partition specific metabolic pathways from the bulk cytoplasm (e.g. Kerfeld *et al.*, 2010). The first bacterial microcompartment (BMC) described was the carboxysome, which is highly active in the fixation of carbon dioxide and encapsulates two proteins: ribulose-1,5-bisphosphate carboxylase/oxygenase and carbonic anhydrase (Shively *et al.*, 1973; So *et al.*, 2004). More recently, BMCs have been associated with other metabolic processes such as 1,2-propanediol catabolism and ethanolamine degradation (Bobik *et al.*, 1999; Kofoid *et al.*, 1999) – pathways that require the activity of more than five different enzymes plus associated cofactors.

BMCs have a polyhedral organization and are assembled from ‘shell proteins’ containing one or two copies of a conserved BMC domain (Pfam ID: PF00936). These proteins are organized as circular hexamers (or pseudohexamers for those with tandem BMC domains) (Crowley *et al.*, 2008; Kerfeld *et al.*, 2005; Sagermann *et al.*, 2009). At least some of the hexameric shell proteins from the different BMCs appear to contain central pores that may facilitate exchange of small metabolites between the compartment and the cytoplasm (Kerfeld *et al.*, 2005; Sagermann *et al.*, 2009). These hexameric arrangements are assumed to form the faces of the polyhedron, with the vertices (Kerfeld *et al.*, 2005) being formed from a pentameric protein of the BMC vertex (BMV) family (Tanaka *et al.*, 2008; Wheatley *et al.*, 2013).

How specific proteins and enzymes are targeted to the interior of a BMC is not fully understood; however, in some cases a short, uncleaved N-terminal signal sequence has been identified on proteins destined for encapsulation (e.g. Fan *et al.*, 2010; Parsons *et al.*, 2010). Recently, it was demonstrated that the targeting sequence of the propionaldehyde dehydrogenase (PduP) protein from *Salmonella enterica* (hereafter *Salmonella*) interacts with a short helix present at the C-terminus of PduA, one of the shell proteins of the BMC dedicated to propanediol utilization (Pdu) (Fan *et al.*, 2012). This finding points to a model whereby proteins are encapsulated into the BMC during its assembly, rather than being targeted into the fully formed BMC post-assembly (Fan *et al.*, 2012).

Since their discovery, BMCs have attracted interest for their potential biotechnological applications (e.g. Frank *et al.*, 2013). The possibility to increase the efficiency, or rate of flux, of a biological pathway through encapsulation, and thus concentration, within a microcompartment remains an attractive one and this would also allow for the shielding of potentially toxic metabolites from the cell cytoplasm. It would be of potentially great interest to design and build a minimalist, empty, synthetic BMC that would be constitutively expressed and adaptable to any number of possible applications. In parallel with the synthetic BMC, compatible protein targeting tools would also have to be designed and tested so that enzymes of choice could be packed within any synthetic BMC so developed. In this study, some progress has been made towards those aims. The *Salmonella pdu* system was used as a template and seven genes from that cluster (*pduA*, -*B*, -*J*, -*K*, -*N*, -*T* and -*U*), which encode the known shell proteins, were assembled into a synthetic operon. The synthetic BMC proteins were shown to be produced in an *Escherichia coli* K-12 chassis, which is one of the current organisms of choice for bioengineering studies (Keasling, 2008; Lee *et al.*, 2008). It was demonstrated that attachment of the N-terminal 20 aa of PduD is sufficient to allow co-purification of heterologous proteins with the protein components of the BMC. Finally, an *in vivo* protease accessibility assay suggests a proportion of a PduD-linked reporter protein is protected from proteolysis when co-expressed with the BMC genes.

## Methods

### 

#### Bacterial strains and growth conditions.

All DNA manipulations were carried out using *E. coli* strain DH5α [Φ80d*lacZ*ΔM15 *recA1 endA1 gyrA96 thi*-*1* *hsdR17*(r_k_^−^ m_k_^+^) *supE44 relA1 deoR* Δ(*lacZYA*-*argF*)*U169*]. For expression of radiolabelled gene products from plasmids under control of the phage T7 ϕ10 promoter, plasmids were transformed into *E. coli* strain K38 (HfrC *phoA4* *pit-10* *tonA22* *ompF627* *relA1*) (Lyons & Zinder, 1972), which carries the compatible plasmid pGP1-2 (Kan^R^) coding for the T7 RNA polymerase (Tabor & Richardson, 1985). Protein purification and fluorescence microscopy were carried out using plasmids transformed into strain MG1655 (F^−^ λ^−^ *ilvG*^−^ *rfb-50* *rph-1*) (Blattner *et al.*, 1997).

#### Plasmid construction.

A list of all the plasmids constructed in this study is provided in Table 1. To amplify the *Salmonella pduAB* genes whilst supplying a 5′-CACAGAGGAACAGGT-3′ linker, which includes an artificial ribosome-binding site (RBS) and a six-base spacer to the initiation codon, at the 5′ end of *pduA* and a hexa-His tag coding sequence to the 3′ end of *pduB*, oligonucleotides PduAB-1 (5′-GCGCTGATCACACAGAGGAACAGGTATGCAACAAGAAGCACTAGGAATGG-3′) and PduAB-2 (5′-GCGCAAGCTTCTGCAGGGATCCTTAGTGATGGTGATGGTGATGGATGTAGGACGGACGATCGTTTTTCGG-3′) were used with *S. enterica* serovar Typhimurium LT2 genomic DNA as template (McClelland *et al.*, 2001). To amplify the *pduJK* pairing, supplying a hexa-His tag coding sequence to the 3′ end of *pduK*, oligonucleotides PduJK-1 (5′-GCGCTGATCACACAGAGGAACAGGTATGAATAACGCACTGGGACTGGTTG-3′) and PduJK-2 (5′-GCGCAAGCTTCTGCAGGGATCCTTAGTGATGGTGATGGTGATGCGCTTCACCTCGCTTGCCGGAATGAATGC-3′) were used. To amplify the *pduN* with a 3′ hexa-His tag coding sequence, oligonucleotides PduN-1 (5′-GCGCTGATCACACAGAGGAACAGGTATGCATCTGGCACGAGTCACGGGCG-3′) and PduN-2 (5′-GCGCAAGCTTCTGCAGGGATCCTTAGTGATGGTGATGGTGATGACACGAAAGCGTATCTACAATGCCG-3′) were used. To amplify the *pduTU* pairing, supplying a hexa-His tag coding sequence to the 3′ end of *pduU*, oligonucleotides PduTU-1 (5′-GCGCTGATCACACAGAGGAACAGGTATGTCTCAGGCTATAGGAATTTTAGAACTCACC-3′) and PduTU-2 (5′-GCGCAAGCTTCTGCAGGGATCCTTAGTGATGGTGATGGTGATGCGTCCGGGTGATCGAGCAAGTGGTG-3′) were utilized. In each case the resultant PCR products were digested with *Bcl*I and *Hin*dIII, and cloned into *Bam*HI–*Hin*dIII-digested pUNI-PROM (Jack *et al.*, 2004) to give plasmids pUNIPROM-AB(Pst), pUNIPROM-JK, pUNIPROM-N and pUNIPROM-TU(Pst), respectively. QuikChange site-directed mutagenesis was then employed to sequentially remove two *Pst*I sites from the coding sequence of *pduB* in plasmid pUNIPROM-AB(Pst) using the primer pairs PduB-pst-1-1/PduB-pst-1-2 (5′-CGGCTATGGCAGAAAAAAGCTGTAGTTTAACGGAATTTGTCGGG-3′; 5′-CCCGACAAATTCCGTTAAACTACAGCTTTTTTCTGCCATAGCCG-3′) and PduB-pst-2-1/PduB-pst-2-2 (5′-GCCGGACATATCGAGCTGCAATACACCGCTCGCGCCAGC-3′; 5′-GCTGGCGCGAGCGGTGTATTGCAGCTCGATATGTCCGGC-3′) to give plasmid pUNIPROM-AB. Site-directed mutagenesis was also used to remove the single *Pst*I site from the coding sequence of *pduU* in plasmid pUNIPROM-TU(Pst) using the primer pair PduU-pst-1/PduU-pst-2 (5′-CTCTTTAAGAAGCTGGGCCTGCAAGATGCAGTGTCCGCCATTGGC-3′; 5′-GCCAATGGCGGACACTGCATCTTGCAGGCCCAGCTTCTTAAAGAG-3′).

**Table 1.** Plasmids utilized in this work

Plasmid	Relevant features	Source
pUNI-PROM	Cloning vector for expression of genes under the control of the *tat* and T7 promoters; Amp^R^	Jack *et al.* (2004)
pUNI-AB(Pst)	Produces PduA and PduB*^His^*; two native *Pst*I restriction sites present in *pduB*	This study
pUNI-AB	As pUNIPROM-AB(Pst) except *Pst*I restriction sites removed from *pduB*	This study
pUNI-JK	Produces PduJ and PduK*^His^*	This study
pUNI-N	Produces PduN*^His^*	This study
pUNI-TU(Pst)	Produces PduT and PduU*^His^*; one native *Pst*I restriction site present in *pduU*	This study
pUNI-TU	As pUNIPROM-TU(Pst) except that *Pst*I site removed from *pduU*	This study
pUNI-ABTU	Produces PduA, PduB*^His^*, PduT and PduU*^His^*	This study
pUNI-ABTUN	Produces PduA, PduB*^His^*, PduT, PduU*^His^* and PduN*^His^*	This study
pUNI-ABTUNJK	Produces PduA, PduB*^His^*, PduT, PduU*^His^*, PduN*^His^*, PduJ and PduK*^His^*	This study
pSU-PROM	Cloning vector for expression of genes under the control of the *tat* promoter; Km^R^	Jack *et al.* (2004)
pSU-D_20_	Encodes the first 20 aa of PduD	This study
pSU-D_40_	Encodes the first 40 aa of PduD	This study
pSU-D_20_-GFP	Encodes the first 20 aa of PduD fused to GFP	This study
pSU-D_20_-mCherry(Pst)	Encodes the first 20 aa of PduD fused to C-terminally HA-tagged mCherry; native *Pst*I restriction site present in *mcherry*	This study
pSU-D_20_-mCherry	As pSUPROM-D_20_-mCherry(Pst) except *Pst*I restriction site removed from *mcherry*	This study
pSU-D_40_-GFP-SsrA	Encodes the first 40 aa of PduD fused to GFP with a C-terminal SsrA tag	This study

To concatenate the cloned *pdu* genes, the *pduTU* genes were amplified using the PduTU-1 and PduTU-2 primers and pUNIPROM-TU as template, digested with *Bcl*I and *Hin*dIII, and cloned into *Bam*HI–*Hin*dIII pUNIPROM-AB, to give pUNIPROM-ABTU. To add *pduN* to this, the *pduN* gene was amplified with PduN-1/PduN-2, digested with *Bcl*I and *Hin*dIII, and cloned into *Bam*HI–*Hin*dIII pUNIPROM-ABTU, to give pUNIPROM-ABTUN. Finally, to add the *pduJK* genes they were amplified with PduJK-1/PduJK2, digested with *Bcl*I and *Hin*dIII, and cloned into *Bam*HI–*Hin*dIII pUNIPROM-ABTUN, to give pUNIPROM-ABTUNJK.

For construction of the plasmids coding for the first 20 or 40 aa of PduD (encompassing the predicted BMC targeting sequence) oligonucleotide PduD-*Bgl*II-For (5′-GCGCAGATCTCACAGAGGAACAGGTATGGAAATTAATGAAAAATTGCTGCGCC-3′), which includes the same 5′-CACAGAGGAACAGGT-3′ RBS and linker employed for the BMC cloning, was used along with either PduD20-rev-Xba (5′-GCGCTCTAGACTTCATATCGCGGAGTACGTCTTC-3′) or PduD40-rev-Xba (5′-GCGCTCTAGAAGCGGTCTGTGGTGCTGTGGATGC-3′) with *Salmonella* chromosomal DNA as PCR template. The resultant PCR products were digested with *Bgl*II and *Xba*I, and separately cloned into *Bam*HI–*Xba*I-digested pSUPROM (Jack *et al.*, 2004) to give pSUPROM-D_20_ and pSUPROM-D_40_, respectively. A gene encoding GFP from plasmid pTGS (DeLisa *et al.*, 2002) was amplified, lacking its initiation codon, with oligonucleotides GFP Primer 1 (5′-GCGCTCTAGAAGTAAAGGAGAAGAACTTTTCACTG-3′) and GFP Primer 2 (5′-GCGCAAGCTTCTGCAGGGATCCTTACGCATAGTCCGGCACATCGTACGGATATTTGTATAGTTCATCCATGCCATGT-3′), digested with *Xba*I and *Hin*dIII, and cloned into similarly digested pSUPROM-D_20_ to give pSUPROM-D_20_-GFP. The same GFP gene encoding a C-terminal SsrA tag was released from plasmid pTGS by digestion with *Xba*I and *Hin*dIII, and cloned into similarly digested pSUPROM-D_40_ to give pSUPROM-D_40_-GFP-SsrA. A gene encoding mCherry, lacking an initiation codon, was amplified with oligonucleotides mCherry Primer 1 (5′-GCGCTCTAGAGTGAGCAAGGGCGAGGAGGATAACA-3′) and mCherry Primer 2 (5′-GCGCAAGCTTCTGCAGGGATCCTTACGCATAGTCCGGCACATCGTACGGATACTTGTACAGCTCGTCCATGCCGCCG-3′), digested with *Xba*I and *Hin*dIII, and cloned into similarly digested pSUPROM-D_20_ to give pSUPROM-D_20_-mCherry(Pst). Subsequently, the native *Pst*I site was removed from the mCherry coding sequence by site-directed mutagenesis using oligonucleotides mCherry *Pst*I site 1/mCherry *Pst*I site 2 (5′-ACCCAGGACTCCTCCCTACAGGACGGCGAGTTCATC-3′; 5′-GATGAACTCGCCGTCCTGTAGGGAGGAGTCCTGGGT-3′). The subsequent plasmid was designated pSUPROM-D_20_-mCherry.

All constructs made during this study were sequenced on both strands to ensure that no undesired mistakes had been introduced during the amplification procedure.

#### Protein methods.

For expression tests of *pdu* genes encoded by plasmids pUNIPROM-AB, pUNIPROM-JK, pUNIPROM-N, pUNIPROM-TU, pUNIPROM-ABTU, pUNIPROM-ABTUN and pUNIPROM-ABTUNJK, which are under control of the phage T7 ϕ10 promoter, each plasmid was used to transform *E. coli* strain K38/pGP1-2 (Tabor & Richardson, 1985). Synthesis of plasmid-encoded gene products was induced by a temperature shift from 30 to 42 °C and followed by labelling with [^35^S]methionine/cysteine mixture as described previously (Coulthurst *et al.*, 2012; Tabor & Richardson, 1985). Samples were separated by SDS-PAGE (12 % w/v acrylamide) after which gels were fixed in 5 % (v/v) acetic acid, 10 % (v/v) methanol, dried and proteins visualized by autoradiography.

Isolation of His-tagged Pdu complexes was carried out by immobilized metal affinity chromatography (IMAC). Briefly, 5–10 l of culture was grown overnight in static 5 l Duran bottles containing LB medium supplemented with 0.4 % (w/v) glucose. Cells were harvested by centrifugation and washed once in 50 mM Tris/HCl (pH 7.5) before being taken up in 25 ml BPER cell lysis cocktail (Thermo Scientific). Lysozyme (0.6 mg ml^−1^) and a crystal of DNase I were added and the suspension was stirred at room temperature for 20 min. Debris and unbroken cells were removed by centrifugation at 20 °C before the supernatant was loaded onto a 5 ml HisTrap FF Crude column (GE Healthcare) equilibrated in 50 mM Tris/HCl (pH 7.5), 200 mM KCl, 50 mM imidazole and 1 mM DDT. Bound proteins were eluted with a 40 ml gradient of 50–1000 mM imidazole in the same buffer.

For electron microscopy, freshly isolated protein was adjusted to a concentration of 0.05 mg ml^−1^ before being applied to carbon-coated copper grids and stained with 2 % (w/v) uranyl acetate. Micrographs were collected at the SULSA Electron Cryomicroscopy Facility at the University of Edinburgh.

SDS-PAGE was performed as described (Laemmli, 1970) and Western blotting was according to Towbin *et al.* (1979). Antisera to the His and haemagglutinin (HA) tags were obtained from Qiagen, and the anti-GFP antibodies were from Life Technologies. Protein identification was performed at cost to the project by Fingerprints Proteomics Service (Dundee).

#### Fluorescence microscopy.

Cells from 1 ml samples of stationary-phase cultures were pelleted by centrifugation, washed with 1 ml of PBS and resuspended in 100 µl of PBS. Cells were then fixed by addition of 150 ml of 4 % (v/v) paraformaldehyde (in PBS) and incubated at room temperature for 10 min. Cells were the pelleted by centrifugation, washed twice, resuspended in 1 ml of PBS and mounted in Hydromount (National Diagnostics). The cells were imaged on a Zeiss LSM 700 laser scanning microscope using a ×100 Plan-Apochromat objective (numerical aperture 1.46), an optical section thickness of 0.7 µm and using the settings optimized for EGFP.

## Results

### Predicting the size and geometry of a BMC

In enteric bacteria such as *Salmonella* and *Citrobacter freundii*, well-characterized BMCs are used in propanediol utilization, with the rationale for compartmentalization being the protection of the cell from a potentially toxic aldehyde intermediate (Kerfeld *et al.*, 2010). The other main biochemical role for a BMC is considered to be the concentration of reactants relative to the total cell volume. Simple geometric principles can be used to predict the physical properties of a BMC and to determine its volume. The *Salmonella* Pdu BMC is thought to be broadly similar in structure to the carboxysome (Frank *et al.*, 2013), given the conserved nature of the predicted shell proteins, and thus it is possible that a Pdu-based BMC will assemble to form something close to an icosahedral structure.

A regular icosahedron is a polyhedron composed of 20 pyramids, the bases of which are equilateral triangles. Placing all the vertices of these pyramids at one point (the centre) ensures that the base triangles tile to form the outer surface of the icosahedron. Thus an icosahedron has 20 faces, 30 edges and 12 vertices on its surface. If the length of the edge of a face triangle is denoted by α, for a regular icosahedron there is a simple relationship between α and the radii of the spheres (*R*_i_*_,_*, *R*_m_ and *R*_c_) that, respectively, inscribe (is tangent to all of the faces of a regular icosahedron), pass through the middle of each surface edge or circumscribe (pass through all of the vertices of an regular icosahedron) as follows:


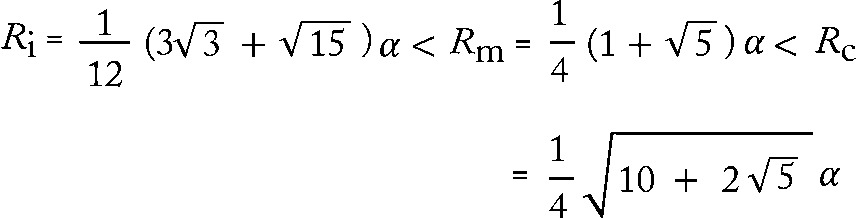


It is known that BMCs vary in size from ~100 to 200 nm across (carboxysomes are reported as ~120 nm across, i.e. ~60 nm in radius) and it is reasonably assumed that the BMC considered here is of a similar size to the carboxysome. These measurements are not sufficiently accurate to be associated precisely with one of the radii defined above. Hence, taking the middle value and setting *R*_m_ = 60 nm yields an edge length α of ~74.16 nm. The volume of the icosahedron, *V*, can therefore be obtained by calculating the volume of a single pyramid and multiplying by the number of pyramids (*n* = 20):


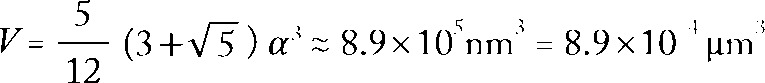


A predicted volume for a BMC of 8.9×10^−4^ μm^3^ is ~1000 times smaller than that of a single cell of the *E. coli* chassis, which has a volume of ~1 µm^3^. This in turn suggests that any reaction that can be housed within a BMC could enjoy an increase in efficiency by several orders of magnitude. Thus, producing a synthetic BMC that could be used to enhance a wide range of biochemical reactions could have broad appeal for biotechnology applications.

### Designing and assembling the components of a synthetic Pdu microcompartment

**Fig. 1.**
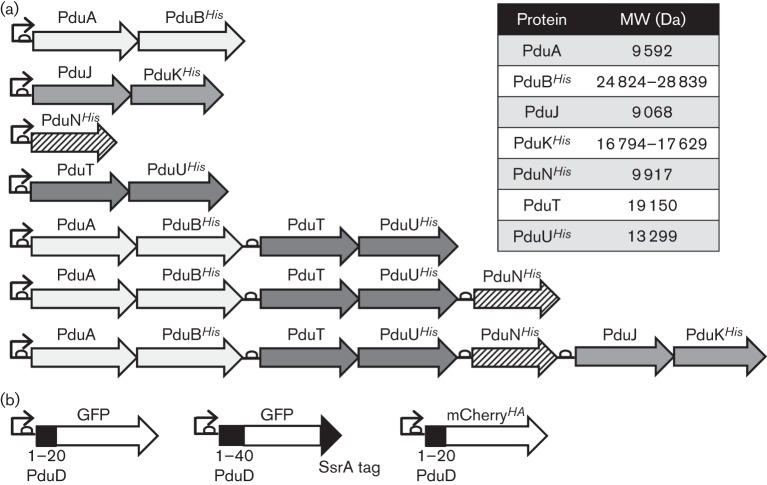
Design of a synthetic operon encoding a BMC and a fluorescent reporter system. (a) Cartoons of the constructs used for production of the BMC shell proteins. Transcription can be driven by the constitutive *E. coli tat* promoter or phage T7 promoter (bent arrow). Natural transcriptional and translational coupling is maintained for *pduAB*, *pduJK* and *pduTU*. The positions of synthetic RBSs are indicated by ovals. The respective names of the gene products are indicated above the arrows and the inset table gives the predicted molecular masses (MW) of those proteins. The *pduB* gene is known to be translated with two alternative initiation sites, one of which overlaps with the *pduA* stop codon whilst the other is in-frame but 111 bp downstream (Havemann & Bobik, 2003). Similarly, the *pduK* gene has two possible translation initiation sites. An AUG codon is present 3 bp downstream of the *pduJ* stop codon but, perhaps more likely, is an alternative GUG initiation codon that is found in-frame but 21 bp downstream and is preceded by a plausible RBS. (b) Cartoons of constructs for targeting reporter proteins to the synthetic BMC. Genetic fusions were made to the N-terminus of the PduD protein and proteolysis or epitope tags are included where indicated. Transcription is driven solely by the *E. coli tat* promoter.

In this work it was decided, due to the close evolutionary relationship with the proposed chassis organism *E. coli*, to engineer the Pdu system from *Salmonella*. The entire *Salmonella pdu* gene cluster contains 18 genes; however, previous studies of the *C. freundii* system have established that just five of these proteins (PduA, -B, -J, -K, and -N) are sufficient to build an empty BMC, while a further two (PduT and -U) also have non-essential structural roles (Parsons *et al.*, 2010). Here, a series of constructs were assembled in order to produce these seven shell proteins in *E. coli* (Fig. 1a). In each case the pUNI-PROM plasmid was chosen, which is a derivative of pT7.5 carrying the constitutive *tat* promoter from *E. coli* (Jack *et al.*, 2004), the idea being that the constitutive promoter would allow constant gene expression in any host strain, but that overexpression could be induced in strains carrying the T7 RNA polymerase if so desired. Genes that occurred together as natural transcription units on the *Salmonella* chromosome (*pduAB*, *pduJK* and *pduTU*) were cloned together with engineered RBSs upstream of the 5′ ends and engineered sequences encoding hexa-His affinity tags at the 3′ ends of the bicistronic units (Fig. 1a). The *pduN* gene was cloned in isolation into pUNI-PROM (Fig. 1a). Naturally occurring *Pst*I restriction sites were silently removed in order to comply as far as possible with current standards suggested by the Registry of Standard Biological Parts. Next, progressively larger synthetic operons were built until the final seven-gene operon encoding PduAB*^His^*, PduTU*^His^,* PduN*^His^* and PduJK*^His^* was completed (Fig. 1a).

**Fig. 2.**
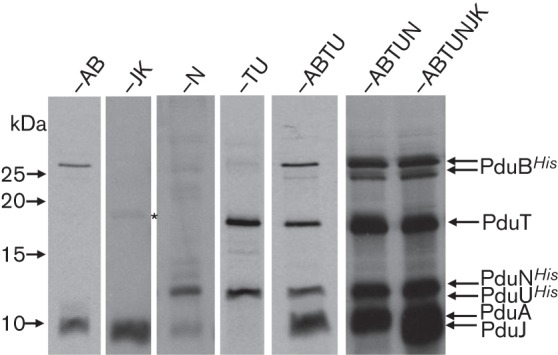
Testing protein production from synthetic constructs in *E. coli*. Total cellular proteins were prepared from small-scale cultures of K38 (pGP1-2) containing the following plasmids: pUNI-AB (‘AB’), pUNI-JK (‘JK’), pUNI-N (‘N’), pUNI-TU (‘TU’), pUNI-ABTU (‘ABTU’), pUNI-ABTUN (‘ABTUN’) and pUNI-ABTUNJK (‘ABTUNJK’). Plasmid-encoded gene products were radiolabelled and separated by SDS-PAGE on a 15 % (w/v) polyacrylamide gel. Protein bands were visualized by autoradiography. The asterisk shows the position of a faint band corresponding to PduK*^His^*.

To test whether each of the seven shell proteins encoded by the bank of *pdu* constructs could be produced stably in an *E. coli* chassis, the phage T7 promoter present on the pUNI-PROM plasmid was exploited. The *E. coli* K-38 (pGP1-2) strain, which produces T7 polymerase, was transformed with the synthetic constructs. Specific labelling of the plasmid-encoded gene products with [^35^S]methionine/cysteine followed by SDS-PAGE and autoradiography revealed clear radiolabelled protein bands for almost all of the Pdu proteins (Fig. 2). Most of the proteins migrated close to their predicted molecular masses (Figs 1a and 2). Upon overexposure of the samples, two forms of the PduB protein were observed, corresponding to two different translation initiation sites, and this has been reported previously (Havemann & Bobik, 2003). The only protein that did not appear to be produced stably in this system was PduK*^His^*, which was visible only as an extremely faint band (Fig. 2).

### PduD–GFP fusion generates bright foci when co-expressed with the BMC components

Having established that the synthetic operon could produce the required BMC shell proteins, the next step was to co-express with potential cargo proteins. A fusion protein was designed whereby the N-terminal 20 aa of the propanediol dehydratase PduD, which during the course of this work have been shown to function as a BMC targeting sequence for this protein (Fan & Bobik, 2011), were fused to a GFP reporter. The PduD_20_–GFP fusion protein was produced under control of the constitutive *tat* promoter from a plasmid with a P15A origin of replication (Bartolomé *et al.*, 1991) and was therefore compatible for co-expression with the pUNI-ABTUNJK construct encoding the BMC.

**Fig. 3.**
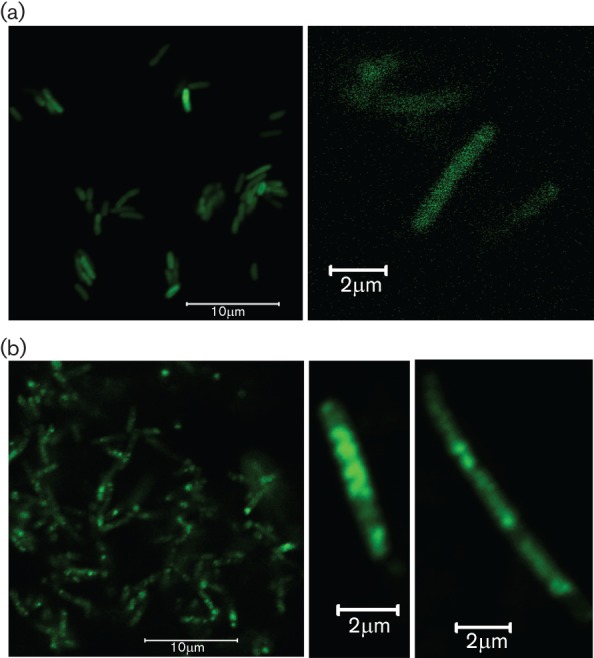
A PduD–GFP fusion generates bright foci when co-expressed with the BMC components. (a) GFP fluorescence signals from *E. coli* MG1655 containing the plasmid pSU-PduD_20_-GFP only. This plasmid encodes a covalent fusion between the initial 20 residues of PduD and the GFP. Left panel: bar, 10 µm; right panel: bar, 2 µm. (b) GFP fluorescence signals from *E. coli* MG1655 co-expressing the plasmid pSU-PduD_20_-GFP together with the plasmid pUNI-ABTUNJK encoding the synthetic BMC. Left panel: bar, 10 µm; centre and right panels: bar, 2 µm.

First, the *E. coli* chassis was transformed with the plasmid encoding the PduD_20_–GFP fusion alone. Confocal microscopy analysis revealed GFP-dependent fluorescence distributed uniformly throughout the cell (Fig. 3a). By contrast, only when PduD_20_–GFP was co-expressed with the synthetic operon encoding the BMC shell proteins did clear foci of GFP fluorescence become visible, consistent with the hypothesis that GFP was being targeted to, or clustered around, a new subcellular structure encoded by the pUNI-ABTUNJK construct (Fig. 3b). It was also notable the BMC-producing cells appeared elongated, perhaps filamentous, in morphology in this co-expression experiment (Fig. 3b). Note that it is not possible to conclude from these data that PduD_20_–GFP is inside a fully formed BMC.

### Cargo proteins co-purify with the shell proteins of the BMC

**Fig. 4.**
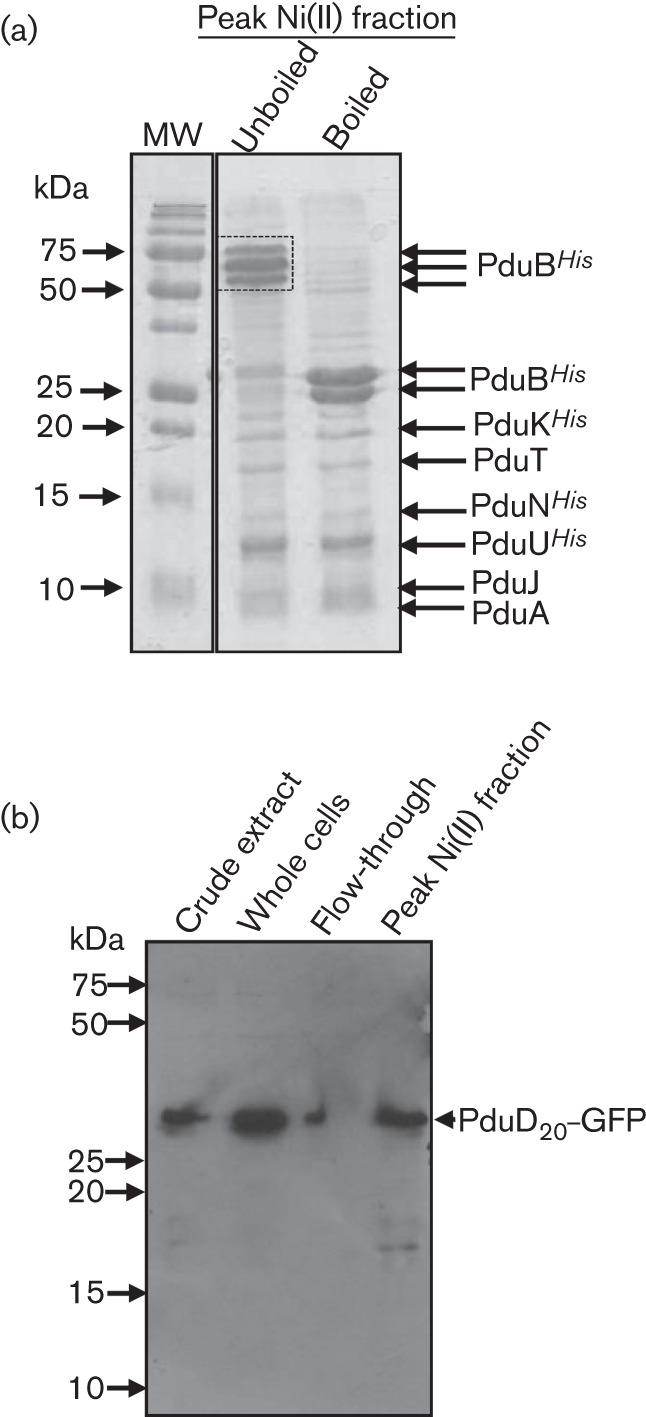
A non-tagged GFP reporter co-purifies with BMC components. *E. coli* MG1655 co-expressing the plasmid pSU-PduD_20_-GFP together with the plasmid pUNI-ABTUNJK encoding the synthetic BMC was anaerobically cultured overnight in LB medium supplemented with 0.4 % (w/v) glucose. Cells were harvested and broken by a chemical cocktail before the crude extract was loaded on to an IMAC column. (a) Protein fractions eluted from the IMAC column were pooled, and analysed by SDS-PAGE and Coomassie staining. Indicated bands were identified by tryptic peptide mass fingerprinting. MW, molecular mass. (b) Pooled fractions from the purification protocol were analysed by SDS-PAGE and Western immunoblotting using an anti-GFP antibody.

Some of the synthetic BMC components (PduB, PduK, PduN and PduU) were engineered with hexa-His affinity/epitope tags (Fig. 1a). This feature was exploited to establish if the PduD_20_–GFP cargo protein was associated with the BMC components in the co-expression experiment (Fig. 3). The *E. coli* chassis was transformed with pUNI-ABTUNJK and pSU-PduD_20_–GFP, and fermented overnight in a glucose-supplemented rich medium. Here, expression of both BMC and cargo was under control of the constitutive *tat* promoter. A crude cell extract was prepared and subjected to IMAC, and proteins were specifically eluted with an increasing imidazole concentration gradient before being further analysed by SDS-PAGE and Coomassie staining (Fig. 4a). Interestingly, if the eluted protein sample was not heat denatured three major protein bands were detectable that migrated with apparent molecular masses between 50 and 75 kDa (Fig. 4a). Tryptic peptide mass fingerprinting analysis of these bands suggested only one *Salmonella* protein was present here and it was PduB*^His^*. However, when the same sample was heated to 100 °C for 2 min before SDS-PAGE the high-molecular-mass PduB*^His^*-containing bands were no longer detectable and were replaced by two clear bands of ~25 kDa, closer to the predicted masses of native PduB*^His^* (Fig. 1a). Tryptic peptide mass fingerprinting confirmed that these 25 kDa bands were indeed PduB*^His^* and also identified two tryptic peptide masses derived from the GFP, giving an initial indication that the reporter had co-purified. The remaining protein bands present in the sample were also identified by tryptic peptide mass fingerprint analysis, which gave unequivocal confirmation that the remaining six Pdu proteins, including PduK, were present in the column fraction. Given the mild, non-denaturing, conditions used throughout this purification protocol, this experiment provides reasonable evidence that the BMC component proteins are soluble within the bacterial cytoplasm.

**Fig. 5.**
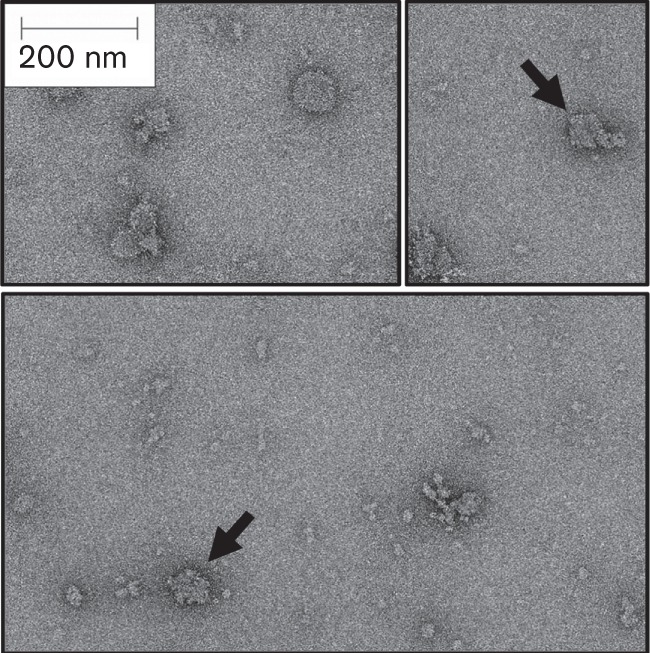
Negative-stain electron microscopy of isolated BMC proteins. Electron micrograph of isolated microcompartment proteins following IMAC. Bar, 200 nm. Particles showing more regular shapes are highlighted by the arrows.

Negative-stain electron microscopy was used to analyse the isolated microcompartment proteins (Fig. 5). The grids showed a range of irregular particles, most of which were <100 nm in size, which is similar to what is expected for native Pdu microcompartments (e.g. Cheng *et al.*, 2008; Sinha *et al.* 2012). A low number of particles adopted more regular shapes (Fig. 5), more akin to carboxysomes isolated from cyanobacteria (Iancu *et al.*, 2007).

In order to localize the PduD_20_–GFP reporter protein in this experiment, the crude cell extract, together with samples of the unbound column flow-through and the peak nickel fraction containing the BMC components (Fig. 4a), was analysed by SDS-PAGE and Western immunoblotting using a GFP-specific antibody (Fig. 4b). Although some GFP was not retained by the metal affinity column and was found in the flow-through fraction (Fig. 4a), a proportion of non-tagged GFP was clearly eluted along with the bound BMC proteins (Fig. 4b).

**Fig. 6.**
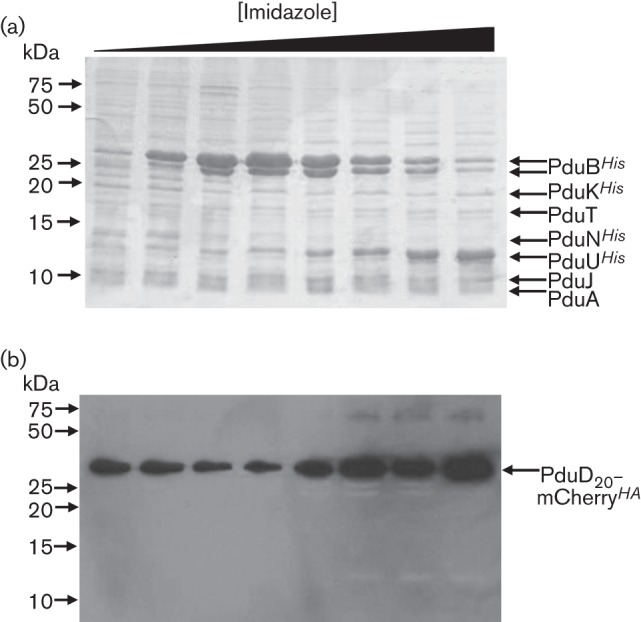
A non-tagged mCherry reporter co-purifies with BMC components. *E. coli* MG1655 co-expressing the plasmid pSU-PduD_20_-mCherry*^HA^* together with the plasmid pUNI-ABTUNJK encoding the synthetic BMC was anaerobically cultured overnight in LB medium supplemented with 0.4 % (w/v) glucose. Cells were harvested and broken by a chemical cocktail before the crude extract was loaded on to an IMAC column and bound proteins eluted with an imidazole gradient. (a) Individual protein peak fractions eluted from the IMAC column during application of an imidazole gradient were collected, boiled, and analysed by SDS-PAGE and Coomassie staining. Indicated bands were identified by tryptic peptide mass fingerprinting. (b) The identical individual protein peak fractions as shown in (a) were analysed by Western immunoblotting using an anti-HA mAb.

To investigate whether another reporter could also be co-purified with the synthetic BMC components, the N-terminal 20 aa of PduD were genetically fused to mCherry that had also been supplied with a C-terminal HA epitope tag for immunodetection. *E. coli* was co-transformed with pSU-D_20_-mCherry*^HA^* and pUNI-ABTUNJK, the culture was fermented with 0.4 % (w/v) glucose, and the BMC proteins were purified by IMAC under non-denaturing, mild conditions. In this case, instead of pooling the fractions that were eluted with increasing imidazole, the fractions across the elution peak were analysed separately by SDS-PAGE and Western immunoblotting (Fig. 6). It is clear that the protein composition varied across the peak, with the earlier fractions containing an excess of PduB*^His^* relative to the other Pdu components (Fig. 6a). Interestingly, PduD_20_-mCherry*^HA^* was immune-detected in all of the fractions eluting from the column (Fig. 6b), but the concentration of antigen was greatest in the fractions that eluted at the highest imidazole concentration, indicative of co-elution of the non-tagged PduD_20_-mCherry*^HA^* with the His-tagged BMC proteins. These data indicate that some of PduD_20_-mCherry*^HA^* is tightly associated with at least one component of the BMC.

### Protease protection assay for assessing BMC function *in vivo*

The evidence presented so far points to the co-expressed PduD fusion proteins being associated with the components of the synthetic BMC. Next, it was important to perform an experiment that could address whether cargo proteins might be located inside the BMC lumen. Thus, an additional construct was prepared where a PduD_40_–GFP fusion was modified at its C-terminus by addition of an SsrA proteolysis tag. Under natural conditions a C-terminal SsrA peptide is added to endogenous *E. coli* polypeptides on stalled ribosomes by the transfer mRNA (Keiler *et al.*, 1996; Komine *et al.*, 1994), which then targets any so-tagged polypeptide for rapid degradation by the ClpAP proteolytic machinery (Karzai *et al.*, 2000).

**Fig. 7.**
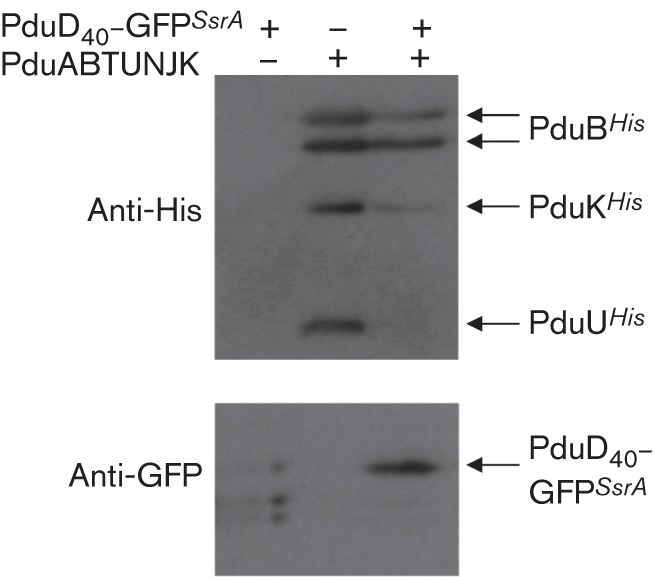
Protease protection assay for assessing BMC function *in vivo*. *E. coli* MG1655 expressing either the plasmid pSU-PduD_40_-GFP*^SsrA^* only, the plasmid pUNI-ABTUNJK encoding the synthetic BMC only or both plasmids together were cultured aerobically in LB medium before being harvested and resuspended in Laemmli disaggregation buffer. The whole-cell samples were then separated by SDS-PAGE and analysed for the presence of His-tagged proteins (the BMC components) or GFP (the cargo protein).

*E. coli* was transformed with plasmids encoding either PduD_40_–GFP*^SsrA^* alone, the BMC shell proteins alone or the PduD_40_–GFP*^SsrA^* fusion protein simultaneously with the shell proteins (Fig. 7). All three strains were grown under identical conditions and analysed by Western immunoblotting (Fig. 7). When PduD_40_–GFP*^SsrA^* was produced in the absence of the BMC proteins the GFP was unstable, presumably because it was rapidly degraded by the ClpAP machinery (Fig. 7). By contrast, the PduD_40_–GFP*^SsrA^* fusion protein was obviously stabilized when it was co-produced with the BMC shell proteins (Fig. 7). This result is consistent with PduD_40_–GFP*^SsrA^* fusion being protected from proteolysis when co-expressed with the BMC genes. One interpretation of these data is that the GFP may be inside the BMC and thus shielded from the cytoplasmic ClpAP system.

## Discussion

The synthetic BMC characterized here was based on the *Salmonella* Pdu system and seven genes from that BMC-encoding gene cluster were organized into a plasmid-borne synthetic operon. All seven gene products were successfully synthesized from a single promoter; however, small-scale expression tests suggested PduK*^His^* was produced at a lower level, or possibly as a more unstable polypeptide, than the others (Fig. 2). The difficulty in detecting radiolabelled PduK*^His^* was surprising as this protein contains two methionines and six cysteines, and so should be expected to radiolabel well in this plasmid-based system. Note, however, that there is an arrangement of four cysteine residues within the C-terminal 30 aa of PduK, which may indicate that the protein binds an iron–sulphur cluster. Indeed, it has been observed that a novel intermolecular iron–sulphur cluster is formed between subunits of another of the Pdu proteins (Parsons *et al.*, 2008). If *Salmonella* PduK were to contain an iron–sulphur cluster then the protein might be destabilized in the small-scale expression system as native *E. coli* transcription is completely inhibited during the labelling procedure, which may affect functionality of the native iron–sulphur cluster assembly machinery. Subsequent larger-scale protein purification experiments, in which host cell biochemistry was allowed to continue uninhibited, readily identified the PduK*^His^* protein (Figs 4 and 6).

Taken together, the GFP and mCherry co-purification experiments described here are consistent with the initial 20 aa of PduD interacting strongly with at least one component of the synthetic BMC. Under mild, non-denaturing condition these non-tagged reporters were seen to co-elute with affinity-tagged shell components. This is consistent with studies of the natural substrates of the *Salmonella* Pdu system (Fan & Bobik, 2011). Recently, the signal sequence of *Salmonella* PduP has been further investigated by alanine scanning mutagenesis, which has identified E7, I10 and L14 of PduP as being important for initial binding to PduA and subsequent encapsulation by the BMC (Fan *et al.*, 2012). Analysis of the PduD N-terminus suggests E5, L8 and I12 would be the analogous important targeting residues in this protein.

The synthetic BMC designed here contains hexa-His affinity tags on four of the seven shell subunits. This allows isolation of all of the BMC components, including those that were not tagged, and some non-tagged cargo proteins. As the PduA, PduJ and PduT proteins were not hexa-His-tagged but also co-purified with the other Pdu proteins following IMAC, it could be concluded that at least some of the native protein–protein interactions are maintained in *E. coli* strains expressing the synthetic pUNI-ABTUNJK construct. One problem with the synthetic system is that it is likely that the plasmid designed here is not producing the shell proteins at the correct physiological stoichiometry. This is evident in the PduD_20_–mCherry*^HA^*/BMC purification experiment where PduD_20_-mCherry*^HA^* was immune-detected in all of the fractions eluting from the column (Fig. 6b), but the concentration of antigen was greatest in the fractions that eluted at the highest imidazole concentration. The later fractions contain less PduB*^His^*, which is produced at the highest levels in this system, but the gel banding profiles later in the gradient are more similar to those observed for authentic Pdu BMCs, which appear to contain PduB at similar stoichiometry to the other shell proteins (Fan *et al.*, 2010; Parsons *et al.*, 2008). It is possible that PduB is being produced in excess here and there is a greater proportion of intact, mCherry-loaded, synthetic BMCs in the later-eluting fractions (Fig. 6). The potential for a range of differently sized protein complexes to be formed, many of which may not be from intact BMCs, is evident in the negatively stained electron microscopy of the isolated BMC components (Fig. 5). Here, a range of differently sized particles and large protein complexes may be present (Fig. 5). The protein purification experiments outlined here do shed some light on the behaviour of PduB*^His^*, as this can be seen to form heat-stable multimers (Fig. 4). Indeed, recent crystallographic analysis suggests this protein does form trimers (Pang *et al.*, 2012), which corroborates this work and at least suggests that individual component proteins are behaving as expected in this heterologous system.

The strongest evidence presented here that a cargo protein may actually be targeted into a BMC expressed from the synthetic operon comes from the protease accessibility assay, where PduD–GFP*^SsrA^* was rescued from destruction by the ClpAP bacterial proteasome by co-expression of the synthetic BMC operon (Fig. 7). However, a similar result may be expected if the PduD–GFP*^SsrA^* protein were to form insoluble aggregates and, indeed, the foci observed in the fluorescence microscopy experiment (Fig. 3) could also be interpreted as aggregation. The IMAC co-purification experiments (Figs 4 and 6) allow an argument to be made against inclusion body or aggregate formation, however, as they would be recalcitrant to purification using this non-denaturing technique.

### Potential applications

The mathematical calculations outlined in this work suggest that the internal volume of a BMC could be 1000 times smaller than that of the bacterial cytoplasm. Thus, the local concentrations of any chemical reactants that could be completely housed within a BMC would be up to 1000 higher than in the cytoplasm of the cell. For example, the law of mass action determines that the reaction rate of a simple, second-order reaction is proportional to the product of the concentrations of the two reactants. Thus, if the concentration of both reactants is increased by a factor of 1000, then potentially this yields an increase in reaction rate by a factor of 10^6^. The potential increase in the reaction rate in the BMC is therefore very significant, especially if multiple enzymes in a single pathway can be co-concentrated within a single BMC.

Harnessing an empty BMC and concomitant targeting sequence has great potential for biotechnological applications (Frank *et al.*, 2013). Using a living chassis for *in vivo* applications may help increase the efficiency of certain chemical reactions (e.g. boosting limonene production by encapsulating recombinant limonene synthase with a source of geranyl pyrophosphate may be helpful in bioenergy research). Indeed, in the course of this work a plasmid encoding a PduD_40_–limonene synthase fusion was constructed (Table S1, available in *Microbiology* Online), but not tested for activity here. The synthetic BMC could also be used *in vivo* to concentrate and sequester toxic compounds such as arsenic. In this regard, a plasmid encoding a fusion between PduD_40_ and metallothionein from *Fucus vesiculosus*, which is an excellent arsenic-binding protein (Ngu *et al.*, 2009), has already been constructed (Table S1).

In conclusion, this paper describes the characterization of a synthetic operon encoding BMC shell proteins that can be expressed in an *E. coli* host. With further development, a synthetic BMC has the potential to be packed with a variety of non-native cargo proteins, which may be useful for both *in vivo* and *in vitro* applications.
